# PLA2G6-Associated Neurodegeneration (PLAN): Review of Clinical Phenotypes and Genotypes

**DOI:** 10.3389/fneur.2018.01100

**Published:** 2018-12-18

**Authors:** Yu-pei Guo, Bei-sha Tang, Ji-feng Guo

**Affiliations:** ^1^Center for Brain Disorders Research, Capital Medical University and Beijing Institute of Brain Disorders, Beijing, China; ^2^Department of Neurology, Xiangya Hospital, Central South University, Changsha, China; ^3^National Clinical Research Center for Geriatric Disorders, Xiangya Hospital, Central South University, Changsha, China; ^4^Center for Medical Genetics, Central South University, Changsha, China; ^5^Key Laboratory of Hunan Province in Neurodegenerative Disorders, Central South University, Changsha, China

**Keywords:** PLA2G6, iPLA_2_β, PLAN, INAD, ANAD, DP, AREP

## Abstract

Phospholipase A2 group VI (PLA2G6)-associated neurodegeneration (PLAN) includes a series of neurodegenerative diseases that result from the mutations in *PLA2G6*. PLAN has genetic and clinical heterogeneity, with different mutation sites, mutation types and ethnicities and its clinical phenotype is different. The clinical phenotypes and genotypes of PLAN are closely intertwined and vary widely. *PLA2G6* encodes a group of VIA calcium-independent phospholipase A2 proteins (iPLA_2_β), an enzyme involved in lipid metabolism. According to the age of onset and progressive clinical features, PLAN can be classified into the following subtypes: infantile neuroaxonal dystrophy (INAD), atypical neuroaxonal dystrophy (ANAD) and parkinsonian syndrome which contains adult onset dystonia parkinsonism (DP) and autosomal recessive early-onset parkinsonism (AREP). In this review, we present an overview of PLA2G6-associated neurodegeneration in the context of current research.

## Introduction

PLA2G6-associated neurodegeneration (PLAN) is a complex group of neurodegenerative diseases that result from mutations in a gene known as *PLA2G6*. According to the age of onset and clinical features, PLAN can be mainly classified into four subtypes: infantile neuroaxonal dystrophy (INAD), atypical neuroaxonal dystrophy (ANAD), adult-onset dystonia-parkinsonism (DP) and autosomal recessive early-onset parkinsonism (AREP). The onset of INAD and ANAD occurs in childhood and these diseases manifest as progressive psychomotor deterioration, axial dystonia, spasticity, and ataxia, as well as optic atrophy in some children. Cerebellar cortical atrophy and iron deposition in the globus pallidus and substantia nigra can be detected by Magnetic Resonance Imaging (MRI) in most patients (1, 2). Past research has suggested that PLAN can be classified as neurodegeneration with brain iron accumulation II (NBIA II) (3, 4). However, although there is a phenotypical intersection between NBIA and PLAN, we propose that neither disease can completely include the other. The onset of DP and AREP occurs in adulthood and patients often have normal birth, achieve early age mile-stones and have a normal childhood. Patients with DP or AREP show clinical manifestations of parkinsonian syndrome. These patients are characterized by bradykinesia and tremors with occurrence of dystonia, in addition to cognitive regression as well as gait instability. Some symptoms are similar to those of parkinsonism, but cerebellar cortical atrophy and iron deposition do not occur in these patients. A high clinical variability is exhibited in these phenotypes, but age of onset and clinical manifestations are the main criteria used to make distinctions between the subtypes of PLAN.

In 2006, the *PLA2G6* gene was initially cloned in two unrelated Israeli INAD families, both of which included consanguineous marriages (5). In 2010, for the first time, the *PLA2G6* gene mutation was associated with parkinsonism (6). At present, the pathogenesis of the *PLA2G6* mutation in neurodegenerative diseases remains unclear. Different mutations and even mutations at the same site may cause phenotypic disparities. For example, the presence of pathogenic *PLA2G6* gene mutation sites (p. D331Y) reported in AREP patients can be associated with a 70% decrease in enzyme activity (7), and the (His597fx69) frameshift mutation can cause the activity of enzymes to differ from previous cases (8). Here, we speculate whether different mutations can result in the diversity of enzymatic activity, thus causing different clinical phenotypes. In this review, we demonstrate clinical phenotypes with different genotypes in PLAN and discuss the underlying relationships of these symptoms with evidence from genetic studies, with a primary focus on the clinical manifestations and genotypic features supported by neuropsychology research, neuroimaging and molecular genetics. Finally, we explore the link between phenotypes and genotypes for PLAN in the light of current *PLA2G6* gene research.

## The Clinical Phenotypes Of PLA2G6-Associated Neurodegeneration

The most common phenotype of the *PLA2G6* gene mutation is NBIA II. PLAN mainly includes INAD, ANAD, and two other diseases are present in parkinsonian syndrome, DP and AREP (9). In addition, some patients present sporadic parkinsonism similar to AREP, known as sporadic early-onset parkinsonism (EOP) (10). *PLA2G6* mutations that cause phenotypical clinical characteristics are shown in Table 1. Moreover, these mutations are associated with hypothyroidism, schizophrenia, diabetes and other diseases (11–13). On magnetic resonance images, most patients carrying the *PLA2G6* mutation showed an iron accumulation in the globus pallidus and/or the substantia nigra in T2-weighted images (13, 14). In pathological examinations of individuals with the *PLA2G6* mutation, abnormal α-synuclein proteins and hyperphosphorylation of tau proteins were found, and may progress to become Lewy bodies (LBs), neurofibrillary tangles and neuropil threads (15, 16). Neuronal biopsies of patients' central nervous systems and peripheral nervous systems tissue provided evidence of the presence of axonal distension, swellings and spheroid bodies (17). In other cases, brain tissue sections with Perl's staining showed iron deposition in the globus pallidus or substantia nigra (18), and oculogyric crises were also found in PLAN patients (19). Here, we describe the clinical features of several PLA2G6-associated neurodegenerative diseases.

**Table 1:** Summary of the clinical features in the PLA2G6-associated neurodegeneration.

	**INAD**	**ANAD**	**DP**	**AREP**
Age of onset	6 months to 3-year-old	Early childhood to juvenescent phase	20-40 years old	Below 40 years old
Progression	Rapid	Slower than INAD	Slow	Slow
Initial symptoms	Psychomotor deterioration	Cerebellar ataxia	Parkinsonism	Parkinsonism
Main clinical symptoms	Tetraparesis, truncal hypotonia, limbo dystonia, mental deterioration, cerebellar ataxia, spasticity, optic atrophy, epilepsy	Psychomotor regression, seizure, gait instability, autism, dystonia, dysarthria, eye movement abnormalities, epilepsy	Bradykinesia, tremor, dystonia, gait instability, rigidity, cognitive deterioration, psychiatric symptoms	Bradykinesia, rigidity, tremor
Important signs	Cerebellar ataxia, hypotonia,hyporeflexia, nystagmus, strabismus	Cerebellar ataxia, hypermyotonia	Hypermyotonia, extrapyramidal signs	Hypermyotonia (especially in lower limbs)
Brain MRI/image signs	Brain iron accumulation in the two sides of globus pallidus and substantia nigra, cerebellar atrophy	Brain iron accumulation in the two sides of basal ganglia, cerebellar atrophy	A substantial reduction in dat	A substantial reduction in dat
EEG	Generalized fast rhythms	Abnormal rhythms	None	None
Pathology	Neuroaxonal swellings and spheroid bodies, Lewy body, hyperphosphorylated-tau	The loss of Purkinje cell in cerebellum, hyperphospholipase-tau, Lewy body	None	None
Lifetime	Short	Longer than INAD	Longer	Longer
Treatment	Symptomatic treatment	Symptomatic treatment	Dopaminergic agents	Dopaminergic agents

*Clinical features of the PLAN presented in this review*.

*INAD, Infantile neuroaxonal dystrophy; ANAD, Atypical neuroaxonal dystrophy; DP, adult-onset dystonia-parkinsonism; AREP, autosomal recessive early-onset parkinsonism; EEG, electroencephalogram; DAT, dopamine transporter*.

### Infantile Neuroaxonal Dystrophy (INAD)

INAD was first discovered and described by Seitelberger in 1952, and was initially known as Seitelberger's disease (20). INAD is an autosomal recessive neurodegenerative disease (20). The age of onset is around 2 years old, mostly occurring before the age of 18 (21). Before the onset of the disease, compared to normal infants, some patients may present a delay in psychomotor development, while most cases present no indication (22). This rare neurological disease is mainly characterized by progressive psychomotor deterioration, truncal hypotonia, cerebellar ataxia, extrapyramidal signs, and early visual failure caused by optic atrophy. Generalized fast rhythms are frequently observed in electroencephalogram (EEG) and seizures may also occur (5, 13, 23–25). Patients often show slight psychomotor and dystonia disorders during infancy and childhood. The other clinical manifestations are bilateral limb spasticity, bulbar signs (impaired swallowing and dyspnoea), pendular nystagmus, strabismus, distal contractures, optic atrophy, and hearing impairment (2, 18, 26–30). Cognitive impairment might also be observed in the disease evaluations. In most INAD cases, an MRI shows signs of iron accumulation in the globus pallidus and/or the substantia nigra (21). At the early stages of the disease, the MRI might not detect the iron accumulation, but as the disease progresses, iron accumulation can often be detected by the MRI between the ages of 3 and 25 years old (18). Another typical sign of INAD is a fast progression of cerebellar atrophy, which is shown by the MRI (31). In addition, some MRIs also show thin optic chiasma, signal hyperintensity of the dentate nuclei and white matter, and cerebral cortical atrophy (32). An electromyography (EMG) also shows denervation in the peripheral nervous system (29), and an EEG can reveal the widespread high-amplitude fast activity at 16–22 Hz after 2 years of age (33). Visual evoked potentials (VEPs) and electroretinograms (ERGs) appear normal in the early stage of the disease, followed by an increase in abnormal signs over time (27). In a Chinese population, axonal spheroids were discovered in the biopsy specimens of skin and sural nerves among ten patients with INAD (34). In addition, neuroimaging showed cerebellar atrophy occurring in the early stages of INAD, but not in other late-onset diseases. Most patients with INAD have progressively worsening symptoms throughout infancy and early childhood and have a shorter survival period (2, 34). The main features of the pathology are axon spheroids and vacuoles, which are widely present in the central and peripheral nervous systems. Brain tissue pathology can also reveal the presence of iron deposits in the bilateral basal ganglia and globus pallidus (35), as well as phosphorylated α-synuclein-positive LBs. Phosphorylated tau-positive neurofibrillary tangles can also be found in some cases (16). Currently, there are no effective treatments, only palliative methods that can relieve symptoms and prevent secondary complications.

### Atypical Neuroaxonal Dystrophy (ANAD)

ANAD is another subtype of PLAN, with atypical clinical characteristics. When the onset of the *PLA2G6* mutation occurs later, the phenotype may be atypical. Different from INAD, the age of onset for ANAD ranges from 3 years old to the late teens. Before the onset of clinical symptoms, motor and intelligence development is relatively normal in these patients (16). Patient symptoms include ataxia, rigidity, spasticity, dystonia, and even myoclonic epilepsy. ANAD is also associated with mental impairment and often visual failure. Some patients develop symptoms before 3 years of age, similar to classical INAD, but neurological deterioration during the course of the disease is often delayed (26). In some cases, an MRI can reveal advanced cerebellar atrophy and iron accumulation in the substantia nigra (18, 21), albeit the absence of cerebellar ataxia (36). Iron deposits in the substantia nigra are present in some atypical cases (15, 21), but it is not a universal feature of PLAN. The majority of late-onset cases lack signs of iron accumulation, and MRIs may even appear completely normal. Other cases may show cortical atrophy or white matter changes; for instance, in one study, obvious cerebellar atrophy was detected during head imaging examination and the bilateral basal ganglia showed signs of iron deposition (2). Pathological examination also revealed the loss of the cerebellar Purkinje cells, the deposition of highly phosphorylated tau proteins that formed neurofibrillary tangles and the deposition of phosphorylated α-synuclein that formed LBs (16). Compared to patients with INAD, patients with ANAD present a slower progression and longer survival times (37). The treatment of ANAD is similar to that of INAD.

## Parkinsonian Syndrome In Plan

### Adult-Onset Dystonia-Parkinsonism (DP)

From the perspective of onset age, DP differs from INAD or ANAD, with a much later onset, occurring from 20 to 40 years old, and exhibiting some typical symptoms, including marked cognitive impairment and some parkinsonian manifestations, such as bradykinesia, ataxia, limb tremors, dystonia, dysarthria, and epilepsy (38, 39). Moreover, oculogyric crises are induced by levodopa in some cases (40). In addition to motor disturbances, non-motor symptoms, such as depression and other adolescent-like behavior changes are observed in DP (41). An MRI of patients with DP reveals some abnormal signals from the frontal lobe, corresponding to severe cognitive impairment. Compared with the MRI results observed in patients with INAD, the MRI results of patients with DP exhibit a rate and degree of severity of iron accumulation and cerebellum atrophy, indicating that these features play a minor role in the manifestation of DP (39, 42). The disease progresses rapidly in patients and is effectively treated with levodopa or polyamine receptor agonists. Patients will often temporarily have dyskinesia after treatment with dopa preparations (6, 38, 43).

### Autosomal Recessive Early-Onset Parkinsonism (AREP)

Based on our previous study, the *PLA2G6* gene was confirmed to be associated with AREP (7). Patients with *PLA2G6*-related AREP exhibited tremors and bradykinesia in the lower limbs, postural instability and hypomimia. Additionally, cerebellar ataxia and autonomic dysfunction were recognized in the late stages of the disease. In the MRI, no evidence of iron deposition was found on T2-weighted images. Levodopa treatment can also be beneficial to AREP patients. In recent years, the *PLA2G6* mutation was found to be closely related to sporadic early-onset parkinsonism (EOP) (6). Unlike AREP, the genetic characterization of EOP is sporadic. The age of onset of EOP is around 20 years old. The disease is characterized by extrapyramidal signs, cognitive decline, dystonia, dysarthria/dysphonia, swallowing problems, limb tremors and abnormal gait, which are sensitive to dopaminergic agents. The MRIs of EOP patients may show iron accumulations in the brain but not in all individuals; frontal lobe and general white matter atrophy can also be observed (44). EEG and EMG examinations are normal in some individuals. Epileptic seizures also occur during the progression of the disease. Currently, some researchers believe that the *PLA2G6* mutation is not a major risk factor for Parkinson's disease in Asian populations (45–47). Moreover, patients with EOP are sensitive to dopamine treatment.

In addition to DP and AREP, there is also a new view regarding the link between PLAN and hereditary spastic paraplegia (HSP). Reports from different countries show that some clinical symptoms of patients with PLAN do not solely include those related to parkinsonian syndrome (48, 49). The clinical features of these patients are mental retardation, extrapyramidal symptoms, lower limb spasticity, cerebellar ataxia, and peripheral neuropathy. Thin corpus callosa and iron accumulations can also be found on MRI images (48, 50).

## The Genotypes Of PLA2G6—Associated Neurodegeneration

**Figure 1:**
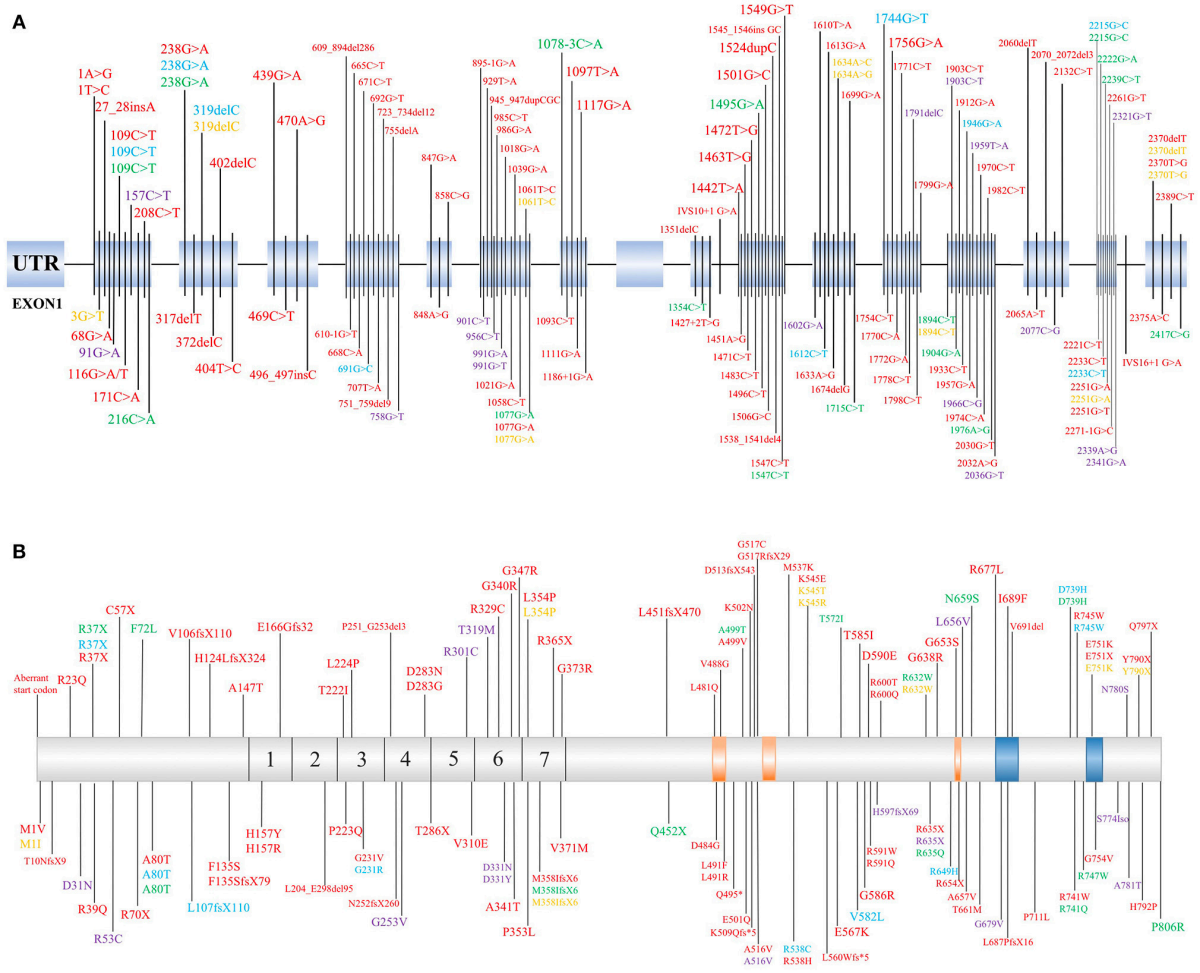
Identified mutation sites of *PLA2G6* gene. **(A)** There are 17 exons of the *PLA2G6* gene, and mutation sites were distributed on the exons. **(B)** Schematic representation in scale of the full-length iPLA2β protein with functional regions and position of the variant protein. The mutations which cause dystonia-parkinsonism, previously identified by others, are shown above. 1–7 = ankyrin repeat region. The position of calmodulin domains and motifs was provided by https://www.uniprot.org/uniprot/O60733. Red, INAD; Blue, ANAD; Green, DP; Yellow, NBIA; Purple, parkinsonian syndrome.

*PLA2G6* mutations have both clinical and genetic heterogeneity. Patients have different types of *PLA2G6* mutations, including missense mutations, truncation mutations, and copy number variations. Individuals carrying the *PLA2G6* mutation can also have different clinical phenotypes depending on specific genotypic features. The previous reported mutation sites of *PLA2G6* are listed in Figure 1 (1, 2, 6–8, 13, 15–18, 22, 23, 30, 34, 38, 41–43, 45, 47, 51–69). Reportedly, in two *PLA2G6* mutation families, all three patients carried *PLA2G6* mutations (p.R632W) but presented different clinical manifestations than previously reported (13, 43). Shi et al. (7) also suggested that the incomplete loss of enzymatic activity causes AREP. Moreover, expansive copy number variants (CNVs) have been detected in the development of PLAN (27, 57). The crystal structure of iPLA_2_β is complex, and the different mutation sites are disparately located on the enzyme (70). According to previous studies, mutation sites in the ankyrin repeat (AR) domains, catalytic(CAT) domains, or any other domains may lead to different enzyme activities. We speculate that the coincident mutation of PLAN may initiate the pathological mechanisms. The pivotal factor that affects the relationships between mutations and clinical phenotypes may be enzyme activity. However, more cases are needed to clarify the relationships between genotypes and phenotypes of *PLA2G6*.

## Genetic Function Of *PLA2G6*

The *PLA2G6* gene is located on 22q13.11 (71), with 17 exons. The protein, encoded by *PLA2G6*, is a member of the A_2_ phospholipase family (PLA_2_), known as group VI calcium-independent phospholipase A_2_ (iPLA_2_β). It is an enzyme functioning in inflammation, immune responses, cell proliferation, apoptosis and remodeling of membrane phospholipids (36, 72).

iPLA_2_β is an intracellular and calcium ion-independent protein. This protein was first isolated from the P388D1 cell line and described in 1994 (73). iPLA_2_β contains 806 amino acids; human iPLA_2_β is 88 KDa and contains an N-terminal domain, ARs domain and CAT domains (70, 74, 75). The iPLA_2_β protein encoded by the *PLA2G6* gene is an important lipase in the human body which is widely distributed in the tissues of human organs (http://www.proteinatlas.org). In the human brain, iPLA_2_β is highly expressed in the substantia nigra, cortex and the hippocampus (76–78). iPLA_2_β can hydrolyse the sn-2 acyl chain of phospholipids and the major decomposition products are docosahexaenoic acid (DHA) and lysophospholipids (75). Under the action of cyclooxygenase and lipoxygenase, DHA produces neuroprotectin D1 (NPD1), and NPD1 plays a crucial role in anti-inflammatory processes and immune responses in the brain (79, 80). NPD1 is specifically involved in the catabolism of fatty acids and arachidonic acid (AA)-related inflammatory reactions (81, 82). iPLA_2_β exerts an anti-inflammatory function through the action of NPD1, against AA, and has a protective effect on cells in inflammatory reactions. The loss of iPLA_2_β's function may affect proteins and processes normally involved in regulating the movement of membranes within axons and dendrites, subsequently leading to mitochondrial abnormalities and synaptic transmission impairment (83–86). Moreover, animal models of PLA2G6 showed neurodegeneration (87) and revealed that iPLA_2_β is closely related to dopaminergic cells, axonal development (88), endoplasmic reticulum stress, mitophagy impairment (89), and changes in Ca(2+) signaling (90). Thus, these animals provide great disease models for PLAN.

iPLA_2_β is associated with a variety of diseases and medical emergencies, including strokes, spinal cord injuries and neurodegenerative diseases (91–93). However, the pathogenesis of *PLA2G6* in neurodegenerative diseases remains unclear and the function of iPLA_2_β, resulting from different mutation sites and types, may be the vital factor. It was reported that pathogenic *PLA2G6* gene mutation sites (p.A341T, p.G517C) in patients with INAD\NBIA can cause significant decreases in enzymatic activity (94). Moreover, the activity of iPLA2β was reduced by 70% compared to normal functioning, owing to the p.D331Y homozygous mutation (7) and the (His597fx69) frameshift mutation, making the activity of enzymes <6% compared to that of WT iPLA_2_β (8). Differential enzymatic activity caused by multifarious mutations may be a key factor in explaining the high clinical variability in PLAN.

## Conclusion

*PLA2G6* mutations have both genotypic and phenotypic heterogeneity. Here, we summarized the major subtypes of PLAN and analyzed their potential relationships. Mutated forms of *PLA2G6* include missense mutations, truncated mutants, fragment deletions, and CNVs. Individuals carrying different *PLA2G6* mutations may also display varied clinical symptoms. The subtypes of *PLA2G6* mutation-related disorders are INAD, ANAD, DP, and AREP, with distinct characteristics associated with each disorder. In recent years, HSP has also been found to be associated with the *PLA2G6* gene mutations. Some mutation sites of HSP also coincide with the mutation sites of the previous four phenotypes. Whether HSP can be considered a PLAN phenotype requires additional research. In the cases of INAD/ANAD, an MRI exhibited iron accumulation in the basal ganglia and globus pallidus, as well as abnormal α-synuclein and hyperphosphorylation of tau proteins in brain tissues, while other cases had relatively moderate MRI features. Moreover, α-synuclein and neurofibrillary tangles pathologies indicate that PLAN may be consistent with idiopathic Parkinson's disease (iPD) to some extent. Interestingly, the view that later onset cases tend to have less tau involvement but still severe α-synuclein pathology may need further discussion. (3, 15, 16, 95).

The link between the phenotypes and genotypes of PLAN suggests that different mutation sites lead to various protein activities. Mutation sites in different domains, for example, ARs or the CAT domains, may have different effects to physiological processes. Based on a previously described case, a *PLA2G6* gene mutation site can result in patients presenting several different phenotypes. Potentially, enzyme activity as well as DNA methylation, synergistic genetic processes, or environmental factors may participate in the pathogenesis of PLAN. Based on the information presented in Figure 1, we speculate that there is no obvious rule for mutation site distribution of PLAN, but further studies are required to clarify why there is no mutation localized in exon 9 and whether the mutations can be categorized according to the 3D structure of iPLA_2_β (70).

In recent decades, the study on *PLA2G6*-related disorders has been performed in terms of pathogenesis and iPLA_2_β function, which provides hope of a detailed understanding of this disease. iPLA_2_β is a vital protein involved in immune responses, inflammatory processes, fatty acid metabolism, oxidative stress, and apoptosis, which may be the pathogenic mechanisms underlying the progression of neurodegenerative diseases. iPLA_2_β is involved in the metabolism of DHA and NPD1, which are closely related to human neurocognitive development and anti-inflammatory properties, respectively (96). Whether mental retardation manifesting in young children is caused as a result of iPLA_2_β affecting the metabolism of DHA is unclear; a better understanding would perhaps provide new information regarding the treatment of PLAN. In addition, iron metabolism may offer clues regarding disease therapy. In summary, the features of PLAN may provide information regarding the etiology of other neurodegenerative diseases.

## Author Contributions

YG conceived the study and wrote the manuscript. BT and JG discussed and revised the manuscript. YG prepared the tables. All authors read and approved the final version of the manuscript.

### Conflict of Interest Statement

The authors declare that the research was conducted in the absence of any commercial or financial relationships that could be construed as a potential conflict of interest.
